# Building a Statewide One Health Network: Report from the Inaugural Pennsylvania One Health Consortium Annual Meeting, 2025

**DOI:** 10.3390/v18050490

**Published:** 2026-04-23

**Authors:** Suresh V. Kuchipudi, Shubhada K. Chothe, Santhamani Ramasamy, James S. Holt, Iulia Vann, Reginald A. Hoyt, Andrew M. Hoffman, Melissa Bilec, Ananias A. Escalante, Jennifer R. Fetter, Sarah States, Maureen Lichtveld

**Affiliations:** 1 School of Public Health, University of Pittsburgh, Pittsburgh, PA 15261, USA; 2 Allegheny County Health Department, Pittsburgh, PA 15219, USA; 3 School of Agriculture and Environmental Sciences, Delaware Valley University, Doylestown, PA 18901, USA; 4 School of Veterinary Medicine, University of Pennsylvania, Philadelphia, PA 19104, USA; 5 Swanson School of Engineering, University of Pittsburgh, Pittsburgh, PA 15261, USA; 6 Institute for Genomics and Evolutionary Medicine, Temple University, Philadelphia, PA 19122, USA; 7 College of Agricultural Sciences, The Pennsylvania State University, University Park, State College, PA 16802, USA; 8 Civil and Environmental Engineering, Carnegie Mellon University, Pittsburgh, PA 15213, USA

**Keywords:** One Health, Pennsylvania, interdisciplinary collaboration, public health infrastructure

## Abstract

The inaugural Pennsylvania One Health Consortium Annual Meeting brought together partners from universities, state agencies, public health, veterinary, agriculture, industry, and community organizations to align around a shared One Health agenda. The program highlighted zoonotic threats, antimicrobial stewardship, wildlife and ecosystem health, invasive species, and climate-sensitive hazards. Participants affirmed privacy-preserving data exchange, projects integrating genomic epidemiology with field and environmental surveillance, cross-disciplinary education, and transparent governance, concluding with a phased roadmap for an integrated statewide One Health framework.

## 1. Introduction

One Health links people, animals, plants, and ecosystems. In Pennsylvania, that linkage is strengthened by a vibrant network of collaborators across academia, government, industry, and communities. The Pennsylvania One Health Consortium (PAOHC) was established to harness these assets into a durable, statewide framework that advances surveillance, preparedness, and response while supporting education and innovation.

The meeting opened with a clear charge from Commonwealth leadership: build on long-standing partnerships to protect health and livelihoods. Keynotes from animal and public-health leaders pair the realities of high-consequence animal diseases and climate-related pressures with a practical vision for prevention grounded in systems thinking, plant and ecosystem health, and community engagement. Scientific sessions then illustrated how Pennsylvania’s partners already work side-by-side: veterinary and public-health teams aligning on antimicrobial stewardship; wildlife and conservation experts integrating health priorities into statewide planning; ecosystem scientists applying life-cycle thinking to sustainability; and educators bringing One Health to students, practitioners, and the public.

This report summarizes how the PAOHC meeting translated shared purpose into a practical path forward. Building on existing strengths across universities, state agencies, and industry partners, Pennsylvania’s statewide One Health network can provide a model for other states and countries to build regional and functional One Health networks.

## 2. Convening Remarks

Russell Redding, Pennsylvania Secretary of Agriculture, opened the inaugural Pennsylvania One Health Consortium Annual Meeting by reflecting on the decade-long journey that has led to this gathering. He emphasized the importance of recognizing the interconnectedness of human, animal, and environmental health and acknowledged key institutions, including the University of Pittsburgh, Pennsylvania State University, the University of Pennsylvania, Delaware Valley University, and Temple University, for their persistent efforts in advancing the One Health vision. He highlighted the organic, cross-sectoral growth of the movement in Pennsylvania, noting that notable progress has been achieved from collective ownership across academia, government, and community partners. Redding described One Health as a systems-based approach that requires collaboration, trust, and long-term commitment. He further celebrated the consortium as a vital step forward in building resilient health systems and closed with a call to action, urging participants to stay committed to the shared labor of building a more integrated, responsive health framework.

## 3. Keynote Address

Dr. Alex Hamberg, Pennsylvania’s State Veterinarian and Director of the Bureau of Animal Health and Diagnostic Services, delivered a keynote presentation titled “The State of Animal Health with Focus on Implications on the Food Supply and Public Health.” He addressed a range of high-impact animal diseases that pose serious threats to agriculture, public health, and food security. He highlighted the growing concern around feral swine as reservoirs for diseases such as pseudorabies and brucellosis, which can be introduced into domestic herds through illegal movement and rescue operations. Dr. Hamberg further discussed African Swine Fever as a significant global threat due to its devastating impact on swine populations and its ability to persist in the environment and spread through global trade and travel. Dr. Hamberg detailed the cascading economic and social consequences of a potential Foot-and-Mouth disease outbreak, referencing historical examples and stressing the mental health toll on farmers. He also discussed the re-emergence of New World screwworm and the challenges of controlling highly pathogenic avian influenza, particularly in dairy cattle. In conclusion, Dr. Hamberg emphasized the urgent need for improved diagnostics, effective communication, and coordinated One Health strategies to protect both animal and human health in the face of emerging threats.

Dr. Maureen Lichtveld, Dean of the School of Public Health at the University of Pittsburgh, delivered a talk titled “One Health as a Disease Prevention Strategy”. She emphasized the critical role of interconnected systems in health, highlighting that food security, especially in the context of global crises, is deeply impacted by environmental disruptions that can lead to mass migration and malnutrition. She stressed that One Health must include not only human, animal, and environmental health, but also plant health, as essential components of a resilient public health framework. Dr. Lichtveld called for translating science into practical interventions across species and sectors and underscored the importance of systems thinking, understanding and responding to the interconnected “systems of systems.” She also highlighted community engagement as a cornerstone of effective One Health strategies. She concluded that true disease prevention and health promotion must be rooted in collective, community-centered approaches that reflect the complexity of today’s ecological and societal challenges.

## 4. Scientific Presentations

**Panel 1: Animal Health and One Health**

Dr. Andrew Hoffman, Dean, School of Veterinary Medicine at the University of Pennsylvania, presented his talk on “The Criticality of Animal Science to One Health”. He emphasized ‘Animal Science’ as an indispensable pillar of One Health, especially in addressing grand global challenges like food insecurity, undernourishment, and food poverty. Drawing on global and regional data, Dr. Hoffman explained how food security remains tightly linked to animal health, especially in low- and middle-income countries where livestock and poultry are central to daily nutrition and economic survival. He highlighted that many food production systems suffer from inefficient yields due to preventable animal diseases such as diarrhea, pneumonia, and reproductive disorders. He stressed that broad food insecurity and poverty cannot be effectively mitigated without addressing endemic livestock health issues. Dr. Hoffman noted that while current One Health strategies focus well on zoonoses and vector-borne diseases, there is an excellent opportunity to better include animal production and welfare in the overall approach to health. He emphasized that integrating animal science into One Health will strengthen public health preparedness and improve sustainable food systems. Dr. Hoffman concluded by advocating for a robust regional and national One Health infrastructure built on veterinary leadership and data-driven decision-making that includes animal production as a core component.

Dr. Erika Ganda, Assistant Professor in the Department of Animal Science at Penn State University, presented an engaging presentation titled “Microbiomes Linking Food Systems, Animals, and Human Health”. Trained as a veterinarian in Brazil, Dr. Ganda’s career has evolved through a global and interdisciplinary lens, spanning animal health, food safety, antimicrobial resistance (AMR), and microbiome-based interventions. She emphasized that as global populations grow and farmland remains finite, microbiome stewardship will be critical to improving agricultural sustainability and public health outcomes. She highlighted *Salmonella dublin* as an example of a One Health challenge, as it is an emerging zoonotic bacterial pathogen capable of causing serious, drug-resistant infections in humans and animals. Through surveillance and genomic analyses, her team found that *S. Dublin* isolates from cattle, humans, and food were often genetically identical, confirming transmission across sectors. Her work revealed alarming AMR patterns, with nearly a third of *S. Dublin* isolates showing resistance to several antibiotic classes. Dr. Ganda closed by advocating for a balanced perspective that promotes integrated, data-driven solutions to shared health threats across species and systems.

Dr. Stephen Cole, Assistant Professor and Director of the Clinical Infectious Disease Laboratory at the University of Pennsylvania, delivered a presentation titled “Bad Bugs and Good Pugs: Antimicrobial Resistance in Companion Animals”, highlighting the growing concern of antimicrobial resistance in pets, particularly the emergence and transmission of carbapenem-resistant Enterobacteriaceae (CRE). These “last resort” drug-resistant bacteria have emerged in pets despite minimal veterinary use of carbapenems, highlighting their potential for silent spread and community transmission. Dr. Cole shared a landmark case of a Great Dane infected with carbapenemase-producing E. coli, which marked the start of an outbreak in a veterinary hospital. Genomic analysis revealed the same resistant strain in other animals, environmental surfaces, and staff, highlighting silent colonization and challenging detection. His lab has since provided infection control consultations to over 20 veterinary hospitals across the U.S. and is leading efforts to track the spread of these organisms using whole-genome sequencing. Dr. Cole advocated for enhanced antimicrobial stewardship, better diagnostic and genomic tools, and the inclusion of veterinary facilities in national AMR surveillance frameworks. He closed by reminding the audience that pets are deeply embedded in human lives and built environments, and therefore must be included in all strategies to address the growing global AMR crisis.

Dr. Julie Ellis, Wildlife Ecologist, Associate Director of the Institute for Infectious and Zoonotic Diseases (IIZD) at Penn Vet, presented “Integrating Fish and Wildlife Health Priorities into the 2025–2035 Pennsylvania Wildlife Action Plan,” highlighting a landmark effort to formally incorporate wildlife health into statewide conservation planning for the first time. Developed in partnership with the Pennsylvania Game Commission and the Fish and Boat Commission, the updated Wildlife Action Plan now includes a comprehensive assessment of health threats to over 600 species of greatest conservation need, including mammals, birds, amphibians, reptiles, fish, and freshwater mussels. Dr. Ellis and her team conducted literature reviews, expert surveys, and interviews to identify key threats, ranging from viruses, fungi, and parasites to industrial pollutants, and wastewater contaminants. These threats are often exacerbated by climate change and disproportionately affect species already facing population pressures. For instance, birds are vulnerable to heavy metals and avian viruses, amphibians face devastating fungal infections like chytridiomycosis, and reptiles are impacted by pesticide exposure and emerging diseases. Dr. Ellis stressed that effective biosecurity practices, including pathogen screening prior to animal translocation, disinfection of field gear, risk assessments, and vaccination strategies are critical tools for mitigation. She also emphasized the need for improved surveillance systems, rapid diagnostic tools, and public outreach to address environmental contaminants and wildlife diseases. Dr. Ellis concluded with a call to give wildlife a full seat at the One Health table, ensuring that environmental and ecological health considerations are prioritized alongside human and animal health in future planning.

**Panel 2: Plant and Ecosystem Health and One Health**

Dr. Melissa Bilec, Special Assistant to the Provost for Sustainability and Co-Director of the Mascaro Center for Sustainable Innovation at the University of Pittsburgh, presented “Integrating Systems Thinking & Sustainable Ecosystems” with a compelling call to adopt holistic frameworks for addressing complex ecological and health challenges. She introduced systems thinking as a vital interdisciplinary approach that enables us to understand relationships among environmental, health, and societal systems rather than viewing components in isolation. Through this lens, she emphasized the importance of circular thinking, building trust across disciplines, and co-developing tools that reflect real-world interconnectedness. A core focus of her talk was Life Cycle Assessment, a quantitative method that evaluates the environmental and health impacts of products and systems from raw material extraction to end-of-life. Dr. Bilec identified key barriers to cross-sectoral collaboration in the One Health space, such as disciplinary silos, persistent data gaps, and misaligned policies, and pointed to opportunities through co-design with affected communities and data-driven policymaking. She urged the One Health community to embrace systems-level tools to anticipate unintended consequences, set measurable sustainability baselines, and advance long-term investments in ecosystem health as preventative health care. Dr. Bilec concluded by encouraging researchers and institutions to shift from isolated work to collaborative partnerships, emphasizing that the complexity of today’s challenges demands integrated, adaptive, and forward-looking approaches.

Dr. Mary Ann Bruns, Professor Emerita of Soil Microbiology at Penn State University, presented “Resilient Soil-Plant-Microbiome Systems for Long-Term Human and Animal Health,” emphasizing that microbiomes are central to ecosystem function and the health of plants, animals, and humans. She explained how plant–soil–microbiome partnerships are essential for land management and agricultural sustainability. Soil microbiomes play a key role in nutrient cycling, pathogen suppression, and improving plant stress tolerance, especially during drought. Bruns highlighted that maintaining soil organic matter is crucial to supporting microbial life, with healthy soils requiring at least 100 times more carbon than the microbial biomass they support. Practices such as conservation tillage, cover cropping, and minimizing physical soil disturbance help protect fungal networks and organic inputs vital to microbial health. These healthy partnerships reduce soil and nutrient loss, improve soil structure, and decrease dependence on synthetic fertilizers. Bruns underscored the risks of ignoring soil health, citing historical and ongoing examples like the Dust Bowl and nutrient-driven dead zones. She concluded by framing soil and microbiome stewardship as one of the core One Health priorities foundational to long-term human, animal, and environmental well-being.

Dr. Deah Lieurance, Assistant Professor of Invasive Species Biology and Management at Penn State, presented “Enhancing Biosecurity by Identifying Invasive Species Threats, Pathways, and Impacts,” emphasizing the critical role invasive species play as one of the top global drivers of biodiversity loss. She underscored their immense ecological and economic impact, citing a global annual cost of over $423 billion, with the U.S. alone incurring nearly $20 billion in damages, primarily in agriculture and forestry. Dr. Lieurance introduced the invasion management curve, illustrating that early detection and rapid response are far more cost-effective than managing widespread invasions. She highlighted how invasive species also intensify wildfire risk. Her research focuses on proactive strategies like horizon scanning and rapid risk assessment to identify high-risk species and prioritize management efforts. She also detailed the importance of pathway management and biosecurity measures, from pre-border cargo inspections and detector dogs to public outreach campaigns like “Don’t Move Firewood.” Dr. Lieurance concluded by advocating for a “One Biosecurity” framework that promotes cross-sector collaboration among human, animal, plant, and environmental health sectors, aligning One Health principles with global and local biosecurity strategies.

Dr. Justin Brown, Assistant Teaching Professor at Penn State University, presented “A One Health Approach to Understanding Disease Risks in Wild and Domestic Canids in Pennsylvania,” focusing on gastrointestinal (GI) parasitism and its implications for animal and human health. He highlighted GI parasites as a persistent challenge in companion animals, noting their potential zoonotic risks despite routine prevention and diagnostics. Dr. Brown introduced the KeyScreen PCR assay as a multiplex pathogen detection tool to detect over 20 GI parasites, including drug-resistant hookworms and zoonotic Giardia strains. His team applied this technology to analyze fecal samples from wild canids (coyotes, foxes), hunting dogs, and companion dogs, generating comparative data across species. Their findings showed significantly higher parasite prevalence in wild canids, including Echinococcus spp. The research revealed that domestic dogs, while not currently positive for Echinococcus, share overlapping ecological spaces and could become reservoirs. Dr. Brown emphasized the importance of bridging communication gaps between veterinary, public health, agriculture, and wildlife sectors using comprehensive diagnostic data to inform integrated surveillance and response efforts.

Robin Eng, Wildlife Ecologist with the Pennsylvania Department of Conservation and Natural Resources, delivered a talk titled “Ecological Communities Drive Ecosystem Restoration: Considering Functional Interactions and Holding Space for the Undiscovered.” Eng emphasized that ecosystem restoration is inherently complex, adaptive, and context-specific, requiring flexibility and responsiveness to changing conditions. She described ecological communities as dynamic, interconnected systems where species interact with one another and their environment in both direct and subtle ways. Restoration must be seen as a collaboration with non-human partners such as soil microbes, fungi, plants, and animals, who are active participants in shaping healthy ecosystems. She also introduced the importance of recognizing functional interactions, such as physical habitat structures that support wildlife behaviors or soil microbial processes that enhance nutrient cycling. She encouraged adaptive management that responds to evolving site conditions and emphasized that biodiversity contributes to ecological richness and improves ecosystem stability. Dr. Eng emphasized the importance of making room for the unknown, recognizing that many factors behind ecosystem health are still undiscovered but crucial for long-term resilience.

**Panel 3: Human Health and One Health**

Dr. Maureen Lichtveld, Dean of the School of Public Health at the University of Pittsburgh, presented “One Health as a Precision Public Health Strategy,” emphasizing the need to shift focus from individual genetics to the broader environmental, social, and ecological determinants that account for the majority of public health risk. She stated that ‘precision public health’ requires fine-tuned, local data to inform targeted interventions, and that One Health offers a powerful framework for integrating such data across human, animal, and environmental sectors. She emphasized that community-engaged science, grounded in local knowledge, is essential for building public trust and ensuring culturally relevant and effective public health interventions. Dr. Lichtveld further discussed global case studies, including mercury contamination from gold mining, underscoring the importance of risk-benefit analyses that include cultural, geographic, and social context. She concluded by emphasizing that predictive, preventative approaches grounded in community data and One Health collaboration will be critical to building healthier, more resilient populations while reducing long-term healthcare costs.

Dr. Suresh Kuchipudi, Professor and Chair of the Department of Infectious Diseases and Microbiology at the University of Pittsburgh, presented “Emerging Zoonotic Viruses and Impacts on Human Health,” emphasizing the urgent need for integrated One Health approaches in managing infectious disease threats. He described that pathogen emergence can be affected by factors such as land use changes, climate change, deforestation, urbanization, and population shift. Modern travel and trade further enable pathogens to move across borders in real time, making traditional surveillance approaches inadequate. Using the example of the H5N1 clade 2.3.4.4b virus, Dr. Kuchipudi illustrated how a virus once considered avian-specific is now circulating among marine mammals, dairy cattle, and humans, demonstrating the complexity of multi-host, multi-directional spillover. Dr. Kuchipudi further emphasized that the integrated cross-sectoral collaboration is hindered by institutional silos, fragmented systems, sector-specific funding and training, regulatory complexity, and data privacy concerns. He proposed ‘Precision One Health’ as a data-integrated framework that can potentially use artificial intelligence, cross-species surveillance, and multidisciplinary science to predict, prevent, and control emerging zoonoses.

Dr. Iulia Vann, Director of Allegheny County Health Department, presented “Hot Zones: How Climate Change is Rewriting the Rules of Public Health,” highlighting the urgent need to reframe public health through a climate-informed, One Health lens. She described how climate change is accelerating environmental disruptions such as wildfires, flooding, and vector-borne disease outbreaks, increasing human health risks and disproportionately impacting communities. Dr. Vann advocated for climate-resilient public health strategies that move beyond reactive responses and instead emphasize integrated surveillance systems, green infrastructure, mobile healthcare delivery, and the development of community resilience hubs. She underscored the importance of cross-sector collaboration and embedding One Health principles into urban and regional planning processes to address systemic inequities and prepare for future threats. Vann concluded that although climate change has created “hot zones” of vulnerability, addressing them effectively will require collaborative, equitable, and proactive public health strategies.

Dr. Ananias A. Escalante, Professor of Biology at Temple University, presented “Introducing BRC-Analytics,” a new bioinformatics platform designed to support infectious disease research through a One Health lens. BRC-Analytics is a free (https://brc-analytics.org/), open-access resource that combines NCBI genomic data with the UCSC Genome Browser and the Galaxy analytical platform, powered by the Texas Advanced Computing Center. The platform allows researchers to conduct high-throughput, reproducible genomic analyses across a wide range of pathogens, vectors, and hosts. With a user-friendly graphical interface, BRC-Analytics is accessible to researchers without programming experience and supports complex phylogenomics, pathogen discovery, and population genomics workflows. The platform emphasizes accessibility, transparency, and reproducibility by tracking each analytical step and providing permanent storage (250 GB) and temporary scratch space (1 TB) for computational work. Users maintain control over data privacy, with the option to share or publish workflows and results. Dr. Escalante highlighted the platform’s potential to enhance scientific collaboration, training, and data-driven decision-making in infectious disease surveillance. Supported by the National Institute of Allergy and Infectious Diseases (NIAID) grant, BRC-Analytics offers a robust infrastructure for integrating genomics into One Health research and public health preparedness. Support is available through the Galaxy Training Network and the BRC Analytics core team.

**Panel 4: Education and Community Engagement**

Reginald Hoyt, Associate Professor and Co-Chair of the Department of Animal Biotechnology and Conservation at Delaware Valley University, presented “One Health for All at DelVal,” highlighting the university’s inclusive and campus-wide approach to One Health education and engagement. DelVal has integrated the concept across disciplines through a grassroots effort involving faculty, staff, and students. The DelVal One Health Working Group was formed to promote awareness within the sciences, humanities, and professional programs. All students are introduced to the concept during their first-year experience, with further exposure through lectures, course assignments, and creative projects such as interviews and public service announcements. DelVal hosts several One Health seminars annually, supports a minor in One Health Communication, and runs immersive experiences, including a mock disease outbreak and exhibits at the campus-wide A-Day Fair. Hoyt emphasized that DelVal’s model prioritizes engagement over formal instruction to make One Health accessible, relatable, and actionable across all fields of study and to the wider public.

Dr. Justin Brown, Assistant Teaching Professor at Penn State University, presented “Integrating One Health: A New Experience in Undergraduate Education at Penn State,” highlighting the university’s growing undergraduate One Health curriculum. The recently introduced One Health minor offers students foundational knowledge through core courses in introductory One Health, epidemiology, and social determinants of health. Beyond the minor, Penn State offers the One Health Scholar Program, a One Health Club, and global learning opportunities such as the Center for Engaged Learning Abroad (CELA) in Belize. Dr. Brown also introduced new undergraduate certificate tracks, focusing on the human–animal interface, climate and environment, and global food security, that allow broader student participation without committing to the full minor. This Penn State initiative is designed to reach students across disciplines and foster meaningful One Health engagement beyond traditional biomedical fields.

Dr. Jennifer Punt, Associate Dean for One Health at the University of Pennsylvania School of Veterinary Medicine, presented “One Health@Penn: Educational Innovations,” highlighting Penn’s growing interdisciplinary ecosystem for One Health education. Her presentation emphasized the integration of disciplines at Penn, including medicine, ecology, botany, biology, engineering, humanities, veterinary medicine and the social sciences into One Health education. Dr. Punt introduced the “One Campus, One Health” certificate program, which blends didactic learning with action-based experiences and cross-disciplinary exposure. Dr. Punt further informed about the VMD plus dual degree programs at Penn that train veterinary students in interdisciplinary problem-solving by combining veterinary education with fields like public health, law, nursing, and environmental studies to address complex One Health challenges. She also emphasized the value of accessible educational tools like the “One Health in One Minute” series and a monthly “Research in Progress/Work in Progress” (RiP/WiP) seminar that fosters dialog across diverse disciplines.

Dr. Sarah States, Director of Research and Science Education at Phipps Conservatory and Botanical Gardens, presented “Gardens as Gateways: Making the Human Connection to One Health,” emphasizing the powerful role of museums and public gardens in fostering connections between people, nature, and health. She highlighted that institutions like Phipps are trusted public resources that can communicate complex issues like climate change, ecosystem health, and One Health in relatable, engaging ways. She described Phipps’ many education and outreach programs, including ‘Homegrown’, a raised-bed gardening initiative for food-insecure communities, as well as youth environmental leadership and climate advocacy programs designed to empower the next generation. These programs center on local solutions and prioritize science communication skills. She concluded that effective One Health work requires empathy, emotional awareness, and inclusive partnerships with trusted community spaces like museums and gardens.

Kyle Van Why, Wildlife Disease Biologist with USDA APHIS Wildlife Services, presented “Role of USDA Wildlife Services and Public Engagement—Wildlife Damage Management, Wildlife Disease, and Emergency Response,” highlighting the agency’s mission to support human–wildlife coexistence through non-regulatory, service-based solutions. His presentation outlined four primary goals of Wildlife Services: to provide wildlife-related services that protect agriculture, natural resources, property, and human health and safety; to develop effective methods and strategies with technology that is biologically, environmentally, and socially sound; to value and invest in people by fostering a culture of innovation; and to inform and communicate with stakeholders and the public. The presentation highlighted two levels of outreach: national and local. National outreach includes developing reports, publications, educational videos, conference presentations, interactive dashboards, and formal training programs. Local outreach focuses on direct technical assistance provided in person or over the phone, as well as presentations, workshops, and field-based interactions with landowners and the public. He underscored the importance of providing accessible, one-on-one communication to ensure people understand agency support and feel empowered to participate. Dr. Van Why concluded by emphasizing that strong public engagement, built on trust and communication, is essential to achieving One Health goals in wildlife management.

Jennifer Fetter, Director of the Center for Agricultural Conservation Assistance Training and Water Resources Program Leader at Penn State Extension, presented “Penn State Extension: Getting the Word Out on One Health Discoveries and Solutions Across Pennsylvania.” Her presentation highlighted how Penn State Extension serves as a statewide resource for translating One Health discoveries into accessible, science-based education and outreach. Dr. Fetter explored the presence of Extension offices in all the PA counties and their participation in biomedicine, food safety, animal science, horticulture, food security, family health, water quality and pest management. Dr. Fetter emphasized that Extension provides resources through newsletters, in-person trainings, online courses, webinars, publications, and direct access to team experts. Extension collects input from strategic partners every 3–5 years to guide content and address emergent issues. Fetter concluded by encouraging engagement through partnerships and collaboration with Penn State Extension.

## 5. Economic Challenges in One Health

Christian Herr, Executive Vice President of PennAg Industries Association, provided a perspective on the economic and emotional costs of animal health emergencies through the lens of One Health. Representing over 450 agribusinesses in Pennsylvania, Herr emphasized that disease outbreaks, such as avian influenza, carry significant financial consequences, with over $1.4 billion in federal spending, including $227 million in direct farm losses and additional investments in diagnostics and monitoring. He highlighted Pennsylvania’s commitment, with over $100 million dedicated to lost income recovery and biosecurity enhancement programs. Herr underscored the value of proactive investment in biosecurity, noting its success in reducing bird losses compared to other states. He further spoke about the emotional toll on farmers, industry workers, and responders involved in mass euthanasia and quarantine operations, describing the long-term distress. Herr also pointed to cultural and policy disconnects that are noted while educating lawmakers and the public about agriculture’s realities. He concluded by advocating for continued research, investment, and rapid response capacity building, emphasizing that economic sustainability and human empathy are essential foundations for advancing One Health success.

## 6. Closing Remarks

Lisa Graybeal, Deputy Secretary for Animal Health and Food Safety at the Pennsylvania Department of Agriculture, delivered closing remarks reflecting personal experience and professional insight into the importance of One Health. Drawing from her dual role as a state leader and third-generation dairy farmer, she underscored the deep interdependence between human, animal, plant, and environmental health. She highlighted the agricultural sector as a frontline example of One Health in practice, where livestock, crops, pollinators, water, and soil health intersect. Graybeal described the far-reaching impacts of highly pathogenic avian influenza in poultry and its unexpected spillover into dairy cattle, noting the resulting economic and public health concerns. She also drew attention to invasive plants and insects, like poison hemlock and the spotted lanternfly, as ongoing threats to agricultural ecosystems. Concluding with a call to action, Graybeal urged continued collaboration across disciplines, reminding attendees that One Health must be rooted in purpose, nurtured through partnership, and grown into a shared framework that strengthens health and resilience across Pennsylvania and beyond.

## 7. Operational Priorities and Next Steps

Several key themes for operationalizing One Health in the state emerged from the conference. First, use a shared data language so clinical, veterinary, wildlife, plant, and environmental signals can be combined securely and interpreted together. Second, launch focused pilots to accelerate cross-disciplinary, multisector research partnerships. Third, invest in people through cross-disciplinary curricula, joint practicums, and community-based training that connect laboratories, farms, clinics, and field sites. Finally, anchor collaboration in transparent governance and prospective evaluation so performance can be tracked and improved over time.

## Data Availability

No new data were created or analyzed in this study. Data sharing is not applicable to this article.

